# Borneo Elephants: A High Priority for Conservation

**DOI:** 10.1371/journal.pbio.0000007

**Published:** 2003-08-18

A new study settles a long-standing dispute about the genesis of an endangered species. With scant fossil evidence supporting a prehistoric presence, scientists could not say for sure where Borneo's elephants came from. Did they descend from ancient prototypes of the Pleistocene era or from modern relatives introduced just 300–500 years ago? That question, as Fernando et al. report in this issue, is no longer subject to debate.

Applying DNA analysis and dating techniques to investigate the elephants' evolutionary path, researchers from the United States, India, and Malaysia, led by Don Melnick of the Center for Environmental Research and Conservation at Columbia, demonstrate that Borneo's elephants are not recent arrivals. They are genetically distinct from other Asian elephants and may have parted ways with their closest Asian cousins when Borneo separated from the mainland, effectively isolating the Borneo elephants some 300,000 years ago.

In the 1950s, Borneo elephants had been classified as a subspecies of Asian elephants (either Indian or Sumatran) based on anatomical differences, such as smaller skull size and tusk variations. This classification was later changed, partly because of the popular view that these animals had descended from imported domesticated elephants. Until now, there was no solid evidence to refute this belief and no reason to prioritize the conservation of Borneo elephants.

Their new status, as revealed by this study, has profound implications for the fate of Borneo's largest mammals. Wild Asian elephant populations are disappearing as expanding human development disrupts their migration routes, depletes their food sources, and destroys their habitat. Recognizing these elephants as native to Borneo makes their conservation a high priority and gives biologists important clues about how to manage them.

**Figure:**
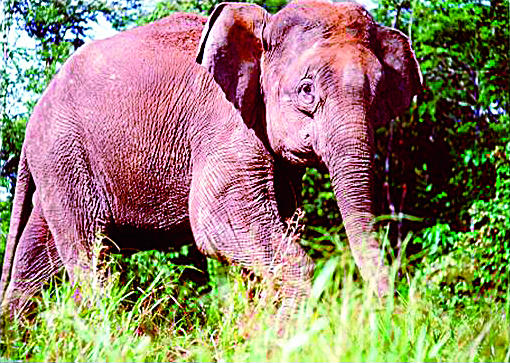
Borneo elephant

